# Supplementary material for: Effects of a Paediatric Antimicrobial Stewardship Program on Antimicrobial Use and Quality of Prescriptions in Patients with Appendix-Related Intraabdominal Infections
Source: Antibiotics (Basel). 2020 Dec 23;10(1):5. doi: 10.3390/antibiotics10010005 (PMC7822420; doi:10.3390/antibiotics10010005)
Supplement: Supplementary file 1 [file antibiotics-10-00005-s001.zip › antibiotics-1036139-supplement/Simó et al Supplementary Tables v2.pdf]

Supplementary Materials: Tables S1-S6

Table S1. Median (IQR) age, in years, according to the different diagnosis of appendix-related intra-abdominal infections (AR-IAI) in the pre-intervention (2014-2016) and in the post-intervention (2017-2019) periods; changes were assessed using the Mann-Whitney U test.

AR-IAI	Pre-intervention period	Post-intervention period	*p*
Phlegmonous appendicitis	10.5 (8.5-13.1)	10.9 (8.9-13.3)	0.316
Gangrenous appendicitis	10.3 (7.8-13.1)	10.5 (8.0-13.0)	0.298
Appendicular peritonitis	8.4 (5.5-11.9)	8.4 (5.4-12.2)	0.337
Appendicular abscess	10.3 (7.8-11.5)	11.0 (9.0-13.0)	0.133
Appendicular mass	9.8 (8.0-11.5)	11.4 (10.1-12.2)	0.359
Total	**10.0 (7.4-12.8)**	**10.4 (7.9-13.0)**	**0.241**

Table S2. Admissions due to appendix-related intra-abdominal infections (AR-IAI) in the pre-intervention (2014-2016) and post-intervention (2017-2019) periods. Changes were assessed using the chi-square test.

**AR-IAI**	**Pre-intervention period (n=919)**	**Post-intervention period (n=1,102)**	***p***
Phlegmonous appendicitis	444 (48.3)	448 (40.7)	<0.001
Gangrenous appendicitis	209 (22.7)	374 (33.9)	
Appendicular peritonitis	243 (26.4)	249 (22.6)	
Appendicular abscess	18 (2.0)	13 (1.2)	
Appendicular mass	5 (0.6)	18 (1.6)	

Table S3. Antimicrobial use (AU) in days of therapy (DOT)/100 patient-days (PD) by drug expressed as median (IQR) of monthly results totalled for each year for better data visualization in appendix-related intrabdominal infections (AR-IAI) during the study period. Drugs with use below 2.0 DOT/100PD in both periods are not shown. Changes were assessed using interrupted time series analysis (step change model).

	**Pre-intervention period**			**Post-intervention period**			***p***
**Antimicrobial (DOT/100 PD)**	**2014**	**2015**	**2016**	**2017**	**2018**	**2019**	
Amoxicillin-clavulanic	2.3 (1.3-3.7)	43.9 (25.8-52.0)	6.3 (5.5-7.6)	3.8 (1.5-6.5)	7.9 (4.2-12.2)	10.9 (6.8-17.0)	0.850
Cefoxitin	49.0 (45.1-58.0)	2.7 (0.0-19.9)	41.6 (34.7-62.6)	54.4 (42.6-64.7)	58.6 (42.2-68.3)	60.7 (54.2-72.4)	0.498
Ceftriaxone	0.0 (0.0-0.0)	0.0 (0.0-0.0)	0.0 (0.0-0.0)	19.1 (0.0-36.8)	27.8 (19.6-32.7)	32.2 (24.2-38.8)	0.816
Meropenem	2.4 (0.0-7.5)	2.0 (0.0-4.7)	2.9 (0.0-10.8)	0.8 (0.0-5.8)	0.0 (0.0-5.1)	2.0 (0.0-4.7)	0.879
Metronidazole	7.6 (0.0-11.8)	1.5 (0.0-3.7)	0.0 (0.0-0.7)	23.7 (0.0-43.5)	32.0 (23.1-45.0)	36.1 (27.5-43.0)	0.449
Piperacillin-tazobactam	53.3 (46.4-59.6)	57.9 (49.6-73.2)	56.1 (47.9-59.2)	11.8 (0.0-62.2)	1.2 (0.0-9.3)	2.2 (0.0-4.3)	0.044
**TOTAL**	**112.7**	**96.4**	**93.7**	**106.8**	**129.4**	**146.2**	**0.113**

Table S4. Antimicrobial use (AU) as length of therapy (LOT, in days) by appendix-related intrabdominal infections (AR-IAI) diagnosis expressed as median (IQR) of monthly results totalled for each year for better data visualization during the study period. Changes were assessed using interrupted time series analysis (step change model).

	**Pre-intervention period**			**Post-intervention period**			***P***
**AR-IAI (days)**	**2014**	**2015**	**2016**	**2017**	**2018**	**2019**	
Phlegmonous appendicitis	3.0 (2.7-3.1)	2.8 (2.6-2.9)	2.7 (2.6-3.0)	1.7 (1.4-1.9)	1.5 (1.3-1.8)	1.5 (1.4-1.6)	<0.001
Gangrenous appendicitis	5.5 (5.0-5.8)	4.6 (4.3-5.8)	5.1 (4.6-5.7)	4.7 (4.5-5.1)	5.2 (4.6-5.6)	4.6 (4.4-4.8)	0.490
Appendicular peritonitis	7.3 (6.9-8.1)	8.2 (6.7-8.9)	8.9 (8.1-9.8)	7.5 (7.0-8.1)	7.7 (6.0-8.5)	8.4 (7.7-9.5)	0.789
Appendicular abscess	0.0 (0.0-6.6)	0.0 (0.0-0.0)	0.0 (0.0-0.0)	0.0 (0.0-0.0)	0.0 (0.0-0.0)	0.0 (0.0-4.3)	0.712
Appendicular mass	0.0 (0.0-0.0)	0.0 (0.0-0.0)	0.0 (0.0-0.0)	0.0 (0.0-0.0)	0.0 (0.0-7.8)	0.0 (0.0-5.5)	0.598
**TOTAL**	**4.7 (4.5-5.2)**	**4.7 (4.4-5.4)**	**5.2 (4.8-5.6)**	**4.5 (4.0-4.6)**	**4.4 (4.0-5.2)**	**4.1 (3.9-4.6)**	0.298

Table S5. Length of stay (LOS, in days) by appendix-related intrabdominal infections (AR-IAI) diagnosis expressed as median (IQR) of monthly results totalled for each year for better data visualization during the study period. Changes were assessed using interrupted time series analysis (step change model).

	**Pre-intervention period**			**Post-intervention period**			***p***
**AR-IAI (days)**	**2014**	**2015**	**2016**	**2017**	**2018**	**2019**	
Phlegmonous appendicitis	2.4<br>(2.3-2.5)	2.3<br>(2.1-2.4)	2.1<br>(2.0-2.5)	1.9<br>(1.7-2.1)	1.8<br>(1.6-1.9)	1.3<br>(1.2-1.4)	0.003
Gangrenous appendicitis	4.7<br>(4.4-5.0)	4.0<br>(3.6-5.3)	4.4<br>(3.5-4.8)	4.0<br>(3.8-4.6)	4.4<br>(4.1-4.8)	3.8<br>(3.5-3.9)	0.448
Appendicular peritonitis	6.9<br>(6.5-7.7)	7.5<br>(6.3-8.3)	8.4<br>(7.5-10.1)	6.7<br>(6.1-7.5)	7.3<br>(6.4-8.1)	7.8<br>(7.1-8.6)	0.822
Appendicular abscess	0.0<br>(0.0-3.6)	0.0<br>(0.0-0.0)	0.0<br>(0.0-0.0)	0.0<br>(0.0-0.0)	0.0<br>(0.0-0.0)	0.0<br>(0.0-3.3)	0.138
Appendicular mass	0.0<br>(0.0-0.0)	0.0<br>(0.0-0.0)	0.0<br>(0.0-0.0)	0.0<br>(0.0-0.0)	0.0<br>(0.0-0.0)	0.0<br>(0.0-3.0)	0.866
**TOTAL**	**4.2**<br>**(4.0-4.7)**	**4.5**<br>**(3.9-5.0)**	**4.6**<br>**(3.8-5.1)**	**4.0**<br>**(3.7-4.5)**	**4.1**<br>**(3.7-4.9)**	**3.6**<br>**(3.3-3.9)**	0.314

Table S6. Antimicrobial stewardship program (ASP) evaluation of antimicrobial prescriptions by appendix-related intrabdominal infections (AR-IAI) diagnosis during the post-intervention period (2017-2019). Changes were assessed using the chi-square test.

	**Optimal**<br>**n (%)**	**Non-optimal**<br>**n (%)**	***p***	**Unknown**<br>**n (%)**
Phlegmonous appendicitis	81 (54.0)	65 (43.3)	<0.001	4 (2.7)
Gangrenous appendicitis	214 (86.3)	32 (12.9)		2 (0.8)
Appendicular peritonitis	235 (91.4)	20 (7.8)		2 (0.8)
Appendicular abscess	35 (77.8)	8 (17.8)		2 (4.4)
Appendicular mass	10 (66.7)	4 (26.7)		1 (6.6)
**TOTAL**	**575 (80.5)**	**129 (18.0)**		**11 (1.5)**
