# Supplementary figures and images for: Effects of a Paediatric Antimicrobial Stewardship Program on Antimicrobial Use and Quality of Prescriptions in Patients with Appendix-Related Intraabdominal Infections
Source: Antibiotics (Basel). 2020 Dec 23;10(1):5. doi: 10.3390/antibiotics10010005 (PMC7822420; doi:10.3390/antibiotics10010005)

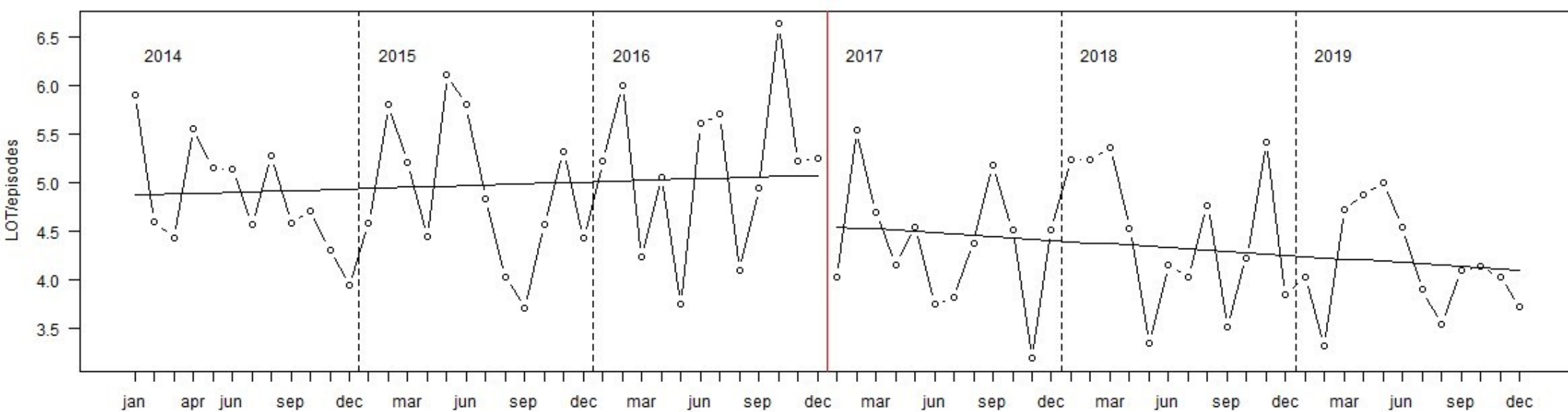

Supplement: Supplementary file 1 [file antibiotics-10-00005-s001.zip › antibiotics-1036139-supplement/Simó et al Figure S1 v1.pdf]

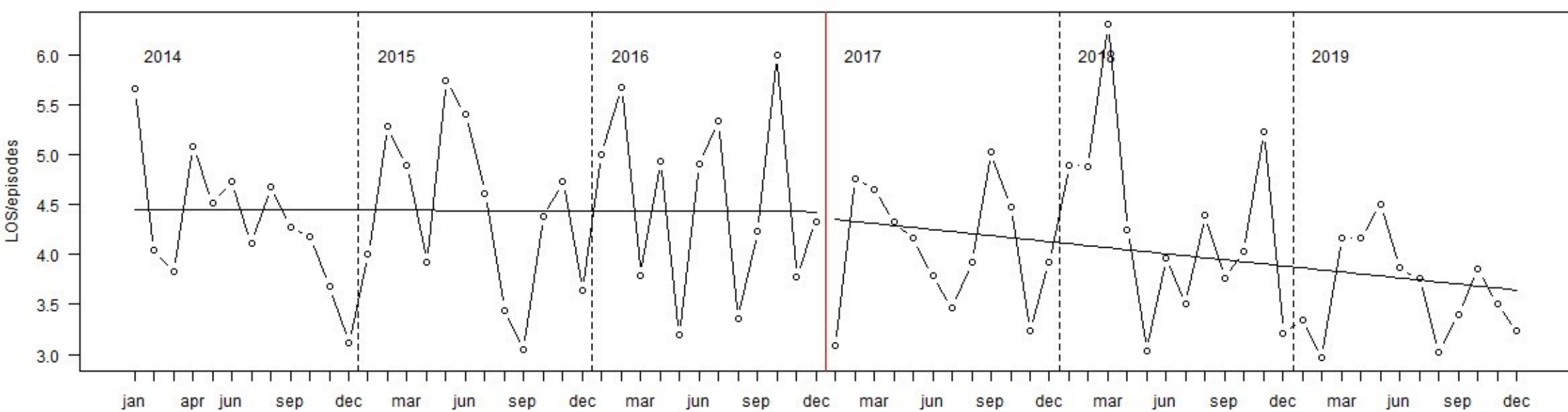

Supplement: Supplementary file 1 [file antibiotics-10-00005-s001.zip › antibiotics-1036139-supplement/Simó et al Figure S2 v1.pdf]

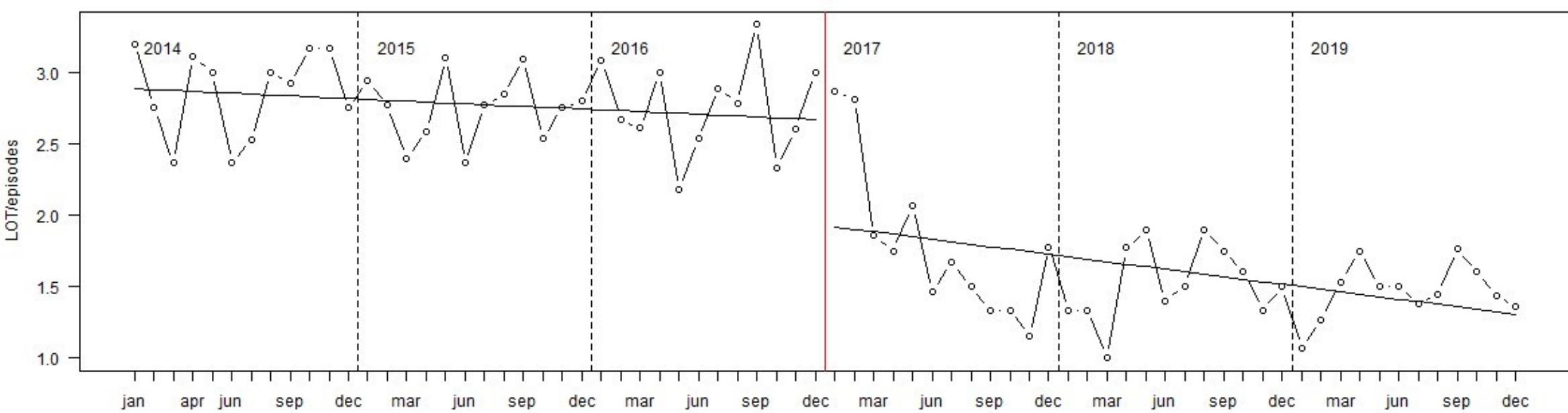

Supplement: Supplementary file 1 [file antibiotics-10-00005-s001.zip › antibiotics-1036139-supplement/Simó et al Figure S3 v1.pdf]

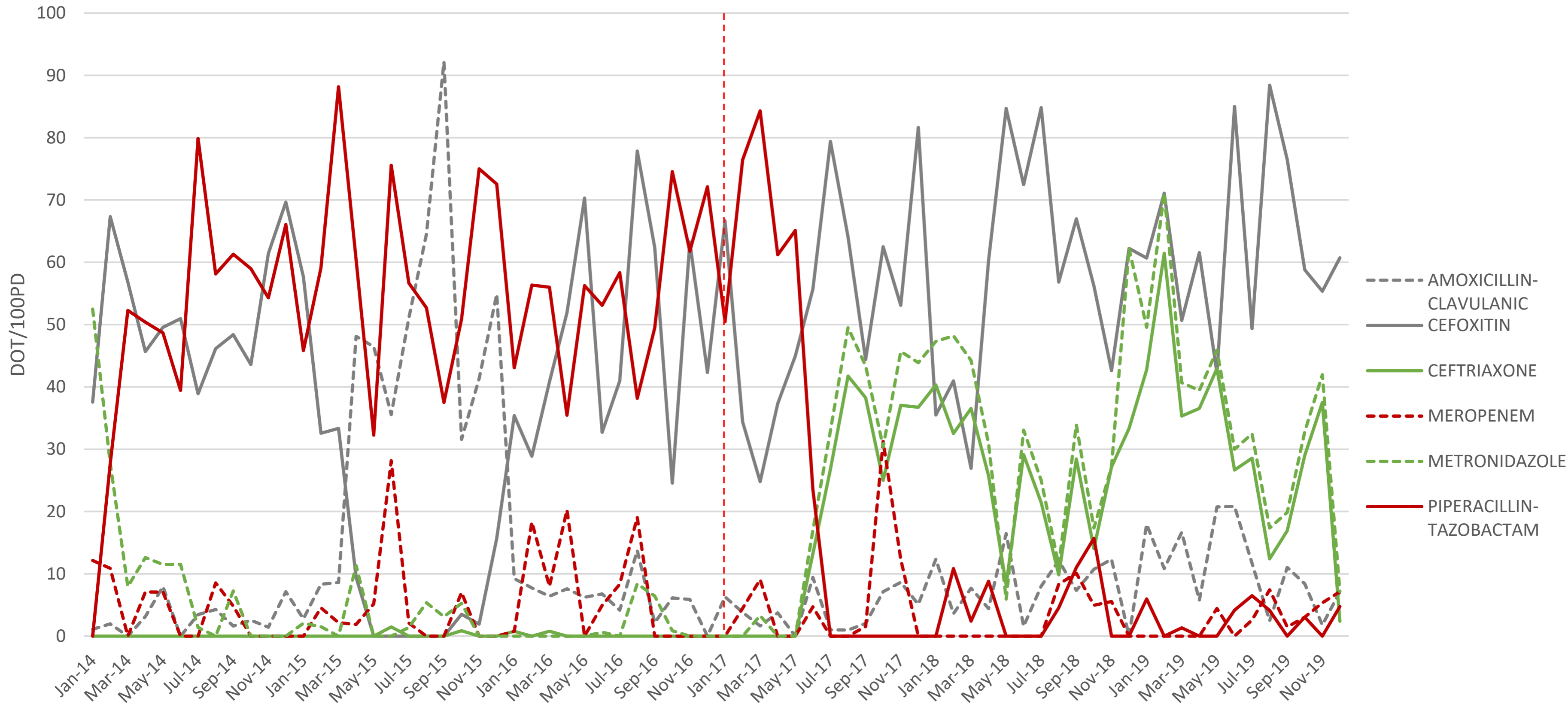

Supplement: Supplementary file 1 [file antibiotics-10-00005-s001.zip › antibiotics-1036139-supplement/Simó et al Figure S4 v1.pdf]
